# Evaluation of ‘care bundles’ for patients with chronic obstructive pulmonary disease (COPD): a multisite study in the UK

**DOI:** 10.1136/bmjresp-2019-000425

**Published:** 2019-05-30

**Authors:** Katherine Morton, Stephanie MacNeill, Emily Sanderson, Padraig Dixon, Anna King, Sue Jenkins, Chris Metcalfe, Ali Shaw, Melanie Chalder, Jonathan Benger, William Hollingworth, James Calvert, Sarah Purdy

**Affiliations:** 1 Population Health Sciences, University of Bristol Faculty of Health Sciences, Bristol, UK; 2 Bristol Medical School, University of Bristol, Bristol, UK; 3 Bristol, UK; 4 Faculty of Health and Life Sciences, University of the West of England, Bristol, UK; 5 University of the West of England, Bristol, UK; 6 Respiratory Medicine, North Bristol NHS Trust, Bristol, UK

**Keywords:** copd exacerbations

## Abstract

**Background:**

Chronic obstructive pulmonary disease (COPD) accounts for 10% of emergency hospital admissions in the UK annually. Nearly 33% of patients are readmitted within 28 days of discharge. We evaluated the effectiveness of implementing standardised packages of care called ‘care bundles’ on COPD readmission, emergency department (ED) attendance, mortality, costs and process of care.

**Methods:**

This is a mixed-methods, controlled before-and-after study with nested case studies. 31 acute hospitals in England and Wales which introduced COPD care bundles (implementation sites) or provided usual care (comparator sites) were recruited and provided monthly aggregate data. 14 sites provided additional individual patient data. Participants were adults admitted with an acute exacerbation of COPD.

**Results:**

There was no evidence that care bundles reduced 28-day COPD readmission rates: OR=1.02 (95% CI 0.83 to 1.26). However, the rate of ED attendance was reduced in implementation sites over and above that in comparator sites (implementation: IRR=0.63 (95% CI 0.56 to 0.71); comparator: IRR=1.12 (95% CI 1.02 to 1.24); group–time interaction p<0.001). At implementation sites, delivery of all bundle elements was higher but was only achieved in 2.2% (admissions bundle) and 7.6% (discharge bundle) of cases. There was no evidence of cost-effectiveness. Staff viewed bundles positively, believing they help standardise practice and facilitate communication between clinicians. However, they lacked skills in change management, leading to inconsistent implementation.

**Discussion:**

COPD care bundles were not effectively implemented in this study. They were associated with a reduced number of subsequent ED attendances, but not with change in readmissions, mortality or reduced costs. This is unsurprising given the low level of bundle uptake in implementation sites, and it remains to be determined if COPD care bundles affect patient care and outcomes when they are effectively implemented.

**Trial registration number:**

ISRCTN13022442.

**Key messages**

- Systematic review evidence from a small number of randomised controlled trials (RCTs) suggested that discharge bundles for patients with chronic obstructive pulmonary disease (COPD) lead to fewer readmissions but do not significantly improve mortality or quality of life. Few data are available on admission bundles for COPD.
- This study found that admission and discharge care bundles for COPD had little impact on patient outcomes, including readmissions and mortality or healthcare costs, but appeared to be associated with a reduced number of subsequent attendances at the emergency department.
- Evaluation of ‘real world’ implementation of admission and discharge care bundles highlighted the challenge of effective implementation of COPD bundles and does not replicate the effectiveness of implementing care bundles demonstrated in RCTs.

## Introduction

Chronic obstructive pulmonary disease (COPD) is one of the most common respiratory diseases in the UK, with an estimated prevalence of 1.2 million people.^1^ Globally, the estimated prevalence of COPD was 251 million cases in 2016, and it is estimated that 3.17 million deaths were caused by the disease in 2015, which is 5% of all deaths.^2^ COPD accounts for 10% of emergency hospital admissions in the UK, and the number of admissions has increased by 50% in the last decade.^1 3^ A third of these patients are readmitted within 28 days of discharge.^3^ COPD admissions are estimated to cost the National Health Service (NHS) £491 million per year.

The care provided for COPD varies across European countries, and an audit in the NHS highlighted wide variation in treatment provision and outcomes for patients admitted for COPD.^4 5^ This disparity was particularly marked in relation to mortality. It also showed that a significant proportion of the observed variability could be explained by access to expert care and evidence-based interventions. There is, therefore, opportunity to improve outcomes for patients with COPD by ensuring that care is consistently provided to a high standard.

The Institute for Healthcare Improvement developed the concept of care bundles with the aim of improving care processes to the highest levels of reliability, which would result in improved outcomes.^6^ A care bundle can be defined as a set of evidence-based clinical interventions or actions which when performed reliably improve patient outcomes. Admission and discharge care bundles for COPD were developed by the British Thoracic Society (BTS) in association with NHS Improvement.^7^ The aim of the admission care bundle is to reduce in-hospital mortality and length of stay, and the discharge care bundle was designed to reduce readmissions. Early pilots in the UK prior to this study suggested possible benefits from care bundles in COPD and other conditions.^8 9^ A single site evaluation of COPD admission care bundles showed delivery of care was improved in the emergency department (ED).^10^ Further studies and a systematic review have focused on discharge bundles, suggesting a reduction in readmissions but highlighting that the impact of care bundles on processes and outcomes of care is poorly understood.^11–14^

This study evaluated the effectiveness of introducing admission and discharge care bundles for patients with an acute exacerbation of COPD (AECOPD) as a means of improving hospital care, and reducing readmissions and mortality, and explored the impact on cost of care and patient and staff experience.

## Methods

### Design

We conducted a mixed-method evaluation using a controlled before-and-after design with nested qualitative case studies to examine the effect of implementing care bundles (online supplementary figure 1). The study was conducted between 2014 and 2017. More details about the study methodology can be found in the published protocol paper.^15^

10.1136/bmjresp-2019-000425.supp1 Supplementary data

### Setting and sampling

We recruited 31 acute hospitals in England and Wales to the study, approaching sites via the BTS and the National Institute for Health Research Clinical Research Network. This included 19 sites which either introduced care bundles during the study period or used care bundles routinely in COPD care (implementation sites), and 12 sites which provided ‘usual care’ throughout the study period (comparator sites). An ‘index date’ was defined for all sites based on when they implemented the bundles—in the case of implementation sites—or the implementation date of a similar site in the comparator sites. Application of a quality improvement (QI) methodology was encouraged in implementation sites throughout the duration of the study using video-conferencing, face-to-face training sessions and one-to-one mentoring.^7^

A subset of these sites provided pseudoanonymised details of all individual patient-level admissions over a period of 12 months preindex and postindex date. With seven pairs of similar implementation and comparator sites, it was estimated that there would be a sample of approximately 10 000 admissions per year. Assuming 30% of patients were readmitted in comparator sites, this would allow us to detect a 9% absolute difference in the COPD readmission rate at 28 days with 90% power and 5% significance level.

### Data collection

Each site was asked to provide data covering a 24-month period—12 months prior to their implementation index date and 12 months afterwards. All sites provided monthly aggregated, routinely collected, Trust-level data for this period (level 1 data). The data included COPD admissions rates, COPD readmission at 28 days and 90 days, mortality, length of stay, ED attendances, and delivery of bundle elements where appropriate. COPD admissions were identified as those with International Classification of Diseases 10th Revision codes J41-44 in the first episode of care.^7^

Sites providing pseudoanonymised patient-level data (level 2) reported outcomes, sociodemographic, clinical and procedure codes plus the information gathered for level 1 sites. Demographic data on individual patients allowed us to study the characteristics of patients admitted with AECOPD and adjust for these in analyses. Each level 2 site was also asked to provide details from the case notes of a random sample of 140 postindex date COPD admissions to provide data on the delivery of the various components of the care bundles (n=2240). Quantitative data submitted to the research team were compiled and checked for both validity and consistency.

Qualitative data were collected from six level 3 sites about the process of bundle implementation or usual care. In addition, data on the context in which bundles were delivered, and the impact on staff, patients and carers, were collected by KM and AK. Methods used to capture data at these four implementation and two comparator sites included document analysis, non-participant observation of patient care, and indepth interviews with health professionals, patients and carers following both admission and discharge, guided by topic guides and observation schedules.

### Care bundle components

The following were the components of the BTS admission bundle:

- A correct diagnosis of AECOPD to be confirmed.
- An oxygen assessment should be undertaken and the correct target range prescribed within 30 min.
- Recognise and respond to respiratory acidosis within 1 hour of admission.
- Medication (steroids and nebulisers) to be administered within 4 hours of admission.
- Review by respiratory team to take place within 24 hours of admission.

The following were the components of the BTS discharge bundle:

- Patients should have respiratory medications and inhaler technique assessed prior to discharge.
- All patients should receive a written plan for how to manage a further AECOPD and should receive a discharge pack of ‘emergency’ drugs prior to discharge.
- Smoking status should be assessed together with a willingness to quit, and for those patients indicating a wish for further assistance a referral should be made to a stop smoking programme.
- All patients should be assessed for their suitability for pulmonary rehabilitation prior to discharge.
- Community follow-up within 2 weeks of discharge from hospital should be organised.

### Data analysis

#### Quantitative analysis of effectiveness data

Trust-level aggregate outcomes were reported monthly for the 12 months before and after their index date. These monthly data were used to calculate the mean change of all outcome measures postindex date at each site. This mean change was then compared between implementation and comparator sites using linear regression analyses on monthly outcomes to estimate how the change postindex date differed between implementation and comparator sites after adjustment for the number of COPD admissions, overall 28-day readmission rate and in-hospital mortality rates in the preindex date period.

The characteristics of patients having at least one COPD admission in the preindex date period were compared between implementation and comparison sites. Linear, logistic and ordered logistic regression models with SEs adjusted for Trust-level clustering were used to test for differences between groups.

Individual-level patient outcomes were studied using a range of multilevel regression models to compare the change in outcomes postindex date using an indicator variable (preindex or postindex date) between the implementation and comparator sites. These analyses controlled for clustering within observations from the same patient and within each Trust using random effects for patient and Trust. Multilevel logistic regression models were used for binary outcomes. Where logistic regression failed to converge, multilevel Poisson models with robust SEs were used. Multilevel negative binomial regression models were used for length of stay and number of emergency admissions due to a large variance in outcome. Models were first run without adjustment for potential confounding, then rerun adjusting for age, sex, ethnicity and Index of Multiple Deprivation quintile. For each logistic regression analysis, ORs and 95% CIs were presented, while for negative binomial and Poisson models the incidence rate ratios (IRRs) and 95% CIs were presented. No formal adjustment was made for multiple testing. Likelihood ratio tests (or Wald tests where robust SEs were used) were used to test for the presence of group–time interaction.

The extent to which sites implemented each element of the bundle was recorded using case note extraction data, and the results were summarised by group using frequencies and proportions. χ^2^ tests were used to compare the proportion of patients receiving bundle elements between implementation and comparator sites.

#### Qualitative analysis

Interviews with staff, patients and carers and observational data from level 3 sites were collected, anonymised and uploaded into NVivo.^16^ All qualitative data were examined using cross-case thematic analysis and analysed both inductively and deductively.^17^ The analysis sought to identify similarities and differences between sites, highlighting aspects which might be transferable to other hospitals intending to implement care bundles. Attention was also given to overlaps or divergence between aspects of practice observed.

#### Quantitative analysis of cost-effectiveness data

An economic evaluation considering the 90-day period following the index admission for COPD was undertaken in level 2 sites. We estimated per**-**patient secondary care NHS costs using healthcare resource group unit costing methodology,^18^ where patient**-**specific resource use was valued using nationally representative sources, for example, NHS reference costs,^19^ the British National Formulary,^20^ and Unit Costs of Health and Social Care.^21^ In the absence of individual-level data on self-reported quality of life or other outcome measures, 90-day mortality following the index admission served as the main outcome measure for the cost-effectiveness analysis. This permits the incremental costs associated with care bundles to be associated with incremental deaths avoided. Cost-effectiveness was calculated as a ratio of the difference in NHS secondary care costs between intervention and comparator sites to the between-site differences in 90-day mortality. Detailed methods are described in the protocol paper^15^ and study report.^22^

### Patient and public involvement

Throughout the 40-month study, the research team conducted a range of patient and public involvement (PPI) activities to ensure that the protocol was properly implemented and that any findings were appropriately interpreted in the light of patient and carer experience. Patients and carers were involved in suggesting the original idea for the study, then commenting on the application for funding, including aims and objectives, methods and PPI. An active PPI group was then formed which continued to provide input on recruitment and patient burden during data collection and study documentation such as consent forms and information sheets. PPI participants also provided feedback on the data available from both qualitative and quantitative sources, commenting on the extent to which it validated their own experiences of care, and provided feedback on possible approaches to the dissemination of the results of the study which would inform patient groups and the wider community.

## Results

### Quantitative findings

#### Analysis of Trust-level aggregate data

Nineteen sites implementing COPD care bundles and 11 comparator sites provided preindex and postindex date data for analysis. One other comparator site was unable to provide data for the full study period. Pooled results are presented in table 1.

**Table 1:** Trust-level outcomes (monthly) pooled across implementation and comparator sites

Outcome	Group	Preindex	Postindex	Difference in the change postindex date between implementation and comparator sites* (95% CI)	P value
Number of COPD admissions, mean (SD)	Comparator	48.02 (21.91)	49.33 (19.33)	0.17 (−6.57 to 6.90)	0.960
	Implementation	52.49 (18.21)	53.93 (17.94)		
28-day COPD readmission rate, mean (SD)	Comparator	11.49 (3.60)	12.79 (4.36)	−1.31 (−5.37 to 2.75)	0.513
	Implementation	15.95 (9.20)	16.07 (11.53)		
28-day overall readmission rate, mean (SD)	Comparator	23.63 (6.70)	24.91 (7.46)	−1.17 (−4.51 to 2.17)	0.478
	Implementation	23.05 (9.90)	23.10 (9.96)		
90-day COPD readmission rate, mean (SD)	Comparator	22.35 (5.59)	23.12 (7.27)	−4.00 (−8.87 to 0.87)	0.103
	Implementation	25.47 (16.42)	22.38 (11.92)		
Number of ED attendances for COPD per month, mean (SD)	Comparator	32.45 (25.17)	37.60 (26.69)	−3.38 (−14.59 to 7.82)	0.525
	Implementation	45.87 (39.27)	47.03 (38.22)		
Length of stay, mean (SD)	Comparator	6.21 (1.96)	5.95 (1.60)	−0.30 (−1.10 to 0.51)	0.453
	Implementation	6.76 (1.36)	6.16 (1.17)		
Total bed days, mean (SD)	Comparator	288.48 (156.30)	275.86 (115.58)	7.83 (−53.66 to 69.32)	0.795
	Implementation	333.15 (121.35)	326.37 (136.62)		

*Adjusted for the number of COPD admissions, in-hospital mortality and overall 28-day readmission for COPD in the preindex date period.

COPD, chronic obstructive pulmonary disease; ED, emergency department.

Implementation sites had a slightly higher mean number of monthly COPD admissions during the preindex date period (52.5 vs 48.0) and a slightly higher mean monthly readmission rate for COPD (15.2% vs 11.5%).

When comparing outcomes for readmissions, ED attendances, length of stay and bed days, the change observed between the preindex and postindex date periods in the implementation sites did not differ from that seen in the comparator sites (table 1). Graphs displaying the monthly 28-day readmission rates for COPD (preindex date) over time by site showed no obvious trends for most sides, although for some there was a clear, strong trend of an increasing admission rate over the year (online supplementary figure 2).

10.1136/bmjresp-2019-000425.supp2 Supplementary data

#### Analysis of individual patient-level data

During the preindex date period, patients in the implementation sites tended to be slightly younger, although the evidence of difference was weak (table 2).

**Table 2:** Characteristics of patients having at least one COPD admission in level 2 implementation and comparator sites at preindex date

Characteristics	Implementation sites	Comparator sites	P value
Number of COPD admissions	4657	4515	
Number of patients having at least one COPD admission	2732	2549	
Age, mean (SD)	71.92 (12.08)	73.02 (11.45)	0.166
Sex, number of male (%)	1351 (49.5)	1240 (48.7)	0.785
Ethnicity, number of patients (%)			
White	2305 (85.59)	2195 (87.7)	0.748
Other	388 (14.4)	307 (12.3)	
Comorbidity, mean Charlson score (SD)	2.07 (1.58)	2.14 (1.65)	0.673
Socioeconomic status, number of patients per IMD quintile (%)			
1 (most deprived)	103 (37.0)	930 (38.2)	0.763
2	552 (20.4)	554 (22.8)	
3	493 (18.2)	506 (20.8)	
4	339 (12.5)	244 (10.0)	
5 (least deprived)	326 (12.0)	198 (8.1)	

COPD, chronic obstructive pulmonary disease; IMD, Index of Multiple Deprivation.

There were 19 097 emergency hospital admissions for COPD during the full study period, of which 13.0% resulted in a readmission for COPD within 28 days. In the preindex date period, 11.6% of COPD admissions had a readmission within 28 days in implementation sites. In comparator sites, the proportion was 14.7%. Postindex date, these proportions were 10.8% in implementation sites but remained at 14.7% in comparator sites. In multivariable regression analyses, there was no evidence that the 28-day readmission rates changed postindex date in either the implementation or comparator sites, and there was no difference in the changes between these two groups (OR for group–time interaction term=1.02 (95% CI 0.83 to 1.26); p=0.865) (table 3).

**Table 3:** Regression model results for primary and secondary outcomes using individual-level data (level 2)

Outcome	Model	COPD admissions in analysis (n)	Change postindex date in comparator sites, estimate (95% CI)	Change postindex date in implementation sites, estimate (95% CI)	Group–time interaction, estimate (95% CI)	P value for interaction term
COPD readmission within 28 days	Unadjusted	19 097	OR=0.97 (0.84 to 1.11)	OR=0.94 (0.81 to 1.10)	OR=0.97 (0.79 to 1.20)	0.804
	Adjusted*	18 324	OR=0.93 (0.81 to 1.07)	OR=0.95 (0.81 to 1.11)	OR=1.02 (0.83 to 1.26)	0.865
All-cause readmission within 28 days	Unadjusted	17 742	OR=1.02 (0.91 to 1.14)	OR=0.86 (0.76 to 0.98)	OR=0.85 (0.71 to 1.01)	0.060
	Adjusted*	16 981	OR=1.00 (0.88 to 1.12)	OR=0.88 (0.77 to 1.00)	OR=0.88 (0.74 to 1.05)	0.156
COPD readmission within 90 days†	Unadjusted	18 381	IRR=0.99 (0.95 to 1.03)	IRR=0.98 (0.84 to 1.15)	IRR=0.99 (0.84 to 1.16)	0.899
	Adjusted*	17 634	IRR=0.98 (0.95 to 1.01)	IRR=0.99 (0.86 to 1.13)	IRR=1.00 (0.87 to 1.16)	0.971
Length of stay for COPD admission	Unadjusted	19 343	IRR=0.99 (0.94 to 1.04)	IRR=0.93 (0.88 to 0.97)	IRR=0.94 (0.88 to 1.01)	0.071
	Adjusted*	18 565	IRR=0.98 (0.94 to 1.03)	IRR=0.93 (0.88 to 0.97)	IRR=0.94 (0.88 to 1.01)	0.100
Number of emergency department attendances	Unadjusted	15 646	IRR=1.14 (1.04 to 1.26)	IRR=0.63 (0.56 to 0.70)	IRR=0.55 (0.47 to 0.63)	<0.001
	Adjusted*	14 900	IRR=1.12 (1.02, 1.24)	IRR=0.63 (0.56 to 0.71)	IRR=0.56 (0.48 to 0.65)	<0.001
In-hospital mortality for COPD admissions	Unadjusted	19 343	OR=0.79 (0.64 to 0.98)	OR=0.79 (0.63 to 0.99)	OR=1.00 (0.74 to 1.36)	0.992
	Adjusted*	18 565	OR=0.78 (0.63 to 0.97)	OR=0.82 (0.65 to 1.03)	OR=1.05 (0.77 to 1.44)	0.741
90-day mortality†	Unadjusted	17 664	IRR=0.93 (0.78 to 1.10)	IRR=0.89 (0.73 to 1.08)	IRR=0.96 (0.74 to 1.24)	0.734
	Adjusted	16 944	IRR=0.93 (0.80 to 1.08)	IRR=0.93 (0.79 to 1.11)	IRR=1.01 (0.80 to 1.26)	0.946

*Adjusted for age, sex, ethnicity and IMD quintile.

†CI calculated using robust SEs; the Wald test was used to generate the p value for interaction.

COPD, chronic obstructive pulmonary disease; IMD, Index of Multiple Deprivation; IRR, incidence rate ratio.

Similar findings were observed for 90-day COPD readmission rates and 90-day mortality (table 3). In the case of 28-day all-cause readmissions, there was weak evidence of a reduction in the implementation sites (OR=0.88 (95% CI 0.77 to 1.00)), but little evidence that this reduction differed from changes observed in the comparator sites (group–time interaction term OR=0.88 (95% CI 0.74 to 1.05); p=0.156) in analyses adjusting for confounders. There was only weak evidence that the reduction in the length of stay in the implementation sites differed from that observed in the comparator sites (group–time interaction term OR=0.94 (95% CI 0.88 to 1.01); p=0.100).

Changes in in-hospital mortality rates did not differ between comparator sites and implementation sites (group–time interaction term OR=1.05 (95% CI 0.77 to 1.44); p=0.741). The number of ED attendances after an initial emergency admission for COPD increased postindex date in the comparator sites (IRR=0.78 (95% CI 0.63 to 0.97)) while the rates dropped in the implementation sites (IRR=0.63 (95% CI 0.56 to 0.71)), and there was evidence that the drop was greater in the implementation sites (group–time interaction term IRR=0.56 (95% CI 0.48 to 0.65); p<0.001).

Sites provided data on care bundle delivery for 1525 patients (77.8% of those requested) (table 4). Delivery of bundle elements generally occurred more frequently in implementation sites than in comparator sites, although few patients in implementation sites received all five BTS-specified bundle elements (admissions bundle: 2.2%; discharge bundle: 7.6% in implementation sites). The average number of admission bundle elements received in comparator sites was 2.2 (SD=1.1) vs 2.6 (SD=1.1) in implementation sites. The average number of individual care elements received on discharge in comparator sites was 1.8 (SD=1.3) vs 2.8 (SD=1.7) in implementation sites (tables 4 and 5).

**Table 4:** Delivery of individual elements of the admissions bundle at level 2 comparator and implementation sites

Admissions bundle				
Bundle element	Delivery	Comparator sites, n (%)	Implementation sites, n (%)	P value
1. Correct diagnosis of AECOPD.				
1a. Chest X-ray result within 4 hours.	Yes	443 (76.8)	454 (83.9)	0.003
1b. ECG result within 4 hours.	Yes	473 (74.8)	656 (83.5)	<0.001
1a and 1b	Yes	350 (59.9)	382 (66.4)	0.022
2. Recognise and respond to respiratory acidosis within 3 hours of diagnosis.				
2a. Arterial blood gas within 1 hour if oxygen sats less than 94% on air or controlled oxygen.	Yes	403 (75.6)	467 (73.8)	0.473
2b. When pH is less than 7.35, assess suitability for Non-invasive ventilation (NIV) and implement within 3 hours of admission.	Yes	345 (94.5)	492 (98.0)	0.006
2a and 2b	Yes	193 (57.4)	283 (61.9)	0.203
3. Recognition of hypoxia and correct oxygen prescription within 30 min of admission with target range of 88%–92%.				
	Yes	197 (32.5)	395 (51.7)	<0.001
4. Correct prescription of medication for AECOPD at admission.				
4a. Steroids prescribed and administered within 4 hours of admission when necessary.	Yes	479 (80.6)	496 (70.2)	<0.001
4b. Antibiotics prescribed and administered within 4 hours of admission when necessary.	Yes	445 (73.4)	535 (74.6)	0.624
4c. Nebulisers prescribed and administered within 1 hour of admission when necessary.	Yes	327 (52.2)	315 (43.5)	0.002
4a, 4b and 4c	Yes	214 (35.8)	207 (29.5)	0.015
5. Review by respiratory specialist (specialist nurse, doctor or physiotherapist) within 24 hours.				
	Yes	100 (17.4)	274 (39.3)	<0.001

AECOPD, acute exacerbation of chronic obstructive pulmonary disease.

**Table 5:** Delivery of individual elements of the discharge bundle at level 2 comparator and implementation sites

Discharge bundle				
Bundle element	Delivery	Comparator sites, n (%)	Implementation sites, n (%)	P value
1. Assess respiratory medications and inhaler technique.				
1a. Respiratory medications.	Yes	342 (53.4)	542 (68.5)	<0.001
1b. Inhaler technique.	Yes	106 (17.1)	307 (40.4)	<0.001
1a and 1b	Yes	102 (16.1)	302 (39.4)	<0.001
2. All patients should receive				
2a. Written pack about managing further AECOPD.	Yes/not applicable	253 (39.6)	404 (51.5)	<0.001
2b. Discharge pack of emergency medications.	Yes/not applicable	170 (26.4)	573 (73.6)	<0.001
2a and 2b	Yes	133 (20.6)	352 (45.3)	<0.001
3. Assess smoking status and willingness to quit.				
	Smoker but cessation not discussed	124 (23.4)	102 (14.4)	<0.001
	Smoker/ex-smoker and cessation discussed	68 (12.8)	173 (24.4)	
	Never smoker/ex-smoker	338 (63.8)	435 (61.3)	
4. Assess for suitability of pulmonary rehabilitation prior to discharge.				
	Yes/completed rehab previously/declined or not applicable	172 (26.4)	407 (51.6)	<0.001
5. Organise community follow-up within 2 weeks of discharge from hospital.				
	Yes, declined or not applicable	356 (54.9)	555 (70.3)	<0.001

AECOPD, acute exacerbation of chronic obstructive pulmonary disease.

### Qualitative findings

Staff perceptions of care bundles were largely positive. Bundles were described as useful for standardising working practices, supporting a clear patient care pathway, facilitating communication between different teams and identifying support required by patients following discharge from hospital. Care bundles were also perceived by staff as a means for embedding reliable, sustainable QI. Staff highlighted the need for managerial support, resourcing and regular training to facilitate QI.

*It means that they [patients] get the care they need, every time, it’s always standard, it’s always how they should be and we know it’s always been done.* (IMP03 ACU6, Lead Nurse, Acute Care)

*with a care bundle, there is a better chance they are going to go out on the right treatment really, particularly if they have not been under the Respiratory Team, and they will have access to more services.* (IMP11 ACU7, ED Consultant)

*I think when patients get discharged I think our checklist that we have works really, really well because it’s a good sort of pointer for us to try and get patients in to see the appropriate people*. (COMP06 ACU3, Respiratory Nurse)

Patients and carers highlighted the need for specialist care and support at the point of discharge, as well as timely follow-up in the community.

*I think I’ll be quite happy and contented as long as I know I’ll be under the COPD nurses.* (IMP05 PAT7, Patient)

The staff data echoed this, by highlighting how pressure around patient numbers, resourcing and staffing in the current context of the NHS can mean that it is not always possible for patients to receive as thorough care, particularly in relation to follow-up, as acute and community staff would prefer. Discharge bundles created opportunities to discuss available services and potential management options.

### Cost-effectiveness findings

COPD care bundles were very unlikely (probability <0.01) to be cost-effective at an arbitrary cost-effectiveness threshold of £20 000 per death avoided at 90 days in a fully adjusted, multiply imputed economic model.

## Discussion

We found no evidence that change in readmissions over time differed between implementation and comparator sites. Furthermore, no difference was observed for in-hospital mortality, length of stay or 90-day readmission. There was a reduction in ED attendances for those receiving care in implementation sites. Additionally, there was a small reduction in the duration of hospital stay for patients in the implementation group. The implementation of care bundles was unlikely to be cost-effective from a secondary care perspective.

Qualitative data suggested that health professionals valued bundles as a means of focusing on standardised quality of care, but staff lacked experience and knowledge in QI strategies. This reflects the findings of Lennox *et al*,^23^ who identify the barriers and facilitators to care bundle implementation. As other authors have identified, the local context, governance structures and financial incentives for support were crucial in determining whether, and how effectively, sites implemented care bundles.^24^ We observed the use of discharge ‘checklists’ at comparator sites, which often represented ‘bundling by another name’. As a result, similar processes and activities seemed to be taking place at implementation and comparator sites. This is likely to have reduced the ability of the study to demonstrate difference in outcomes attributable to care bundles.

To achieve improvements in outcomes, care bundles must meet three criteria: the target outcome must be sensitive to change and responsive to the elements within the bundle; the care bundles must be reliably implemented to ensure that the majority of patients receive bundle-led care; and use of the care bundle must improve process reliability.^13^ In this study the bundles were not delivered reliably, with delivery of the full five-item admission bundle and discharge bundle being very low, although there is some evidence from our data that those patients in receipt of a bundle received more of the required elements of care, suggesting some improvement in process reliability. The third criterion was not met since most patients did not receive the full set of bundle elements. Therefore, effective implementation of the admission and discharge bundles did not occur and the anticipated difference between groups was not observed.

The original BTS COPD care bundles project included QI methodology and continuous data collection to ensure compliance with elements of bundle delivery.^7 13^ However, few sites fully engaged with this process during this study, and it was clear from site visits that staff had insufficient time to change processes of care reliably.

### Strengths and limitations

Due to the timing of care bundle implementation nationally, it was not possible to undertake a randomised controlled trial to evaluate the impact of care bundles. While site recruitment was facilitated through several routes, it is possible that the participating sites were those with a respiratory team who had an interest in care bundles or research activity related to COPD. Thus, they may have demonstrated ‘better’ performance with regard to COPD care and QI than average, introducing recruitment bias to the study.

Conducting research in the context of acute admissions posed challenges to patient recruitment and data collection, particularly in terms of data availability and completeness. Additional limitations of the study include potential reporting bias as some sites were unable to provide useful level 1 and 2 data. There were also differences in patient populations at different sites and implementation sites had higher baseline COPD readmission rates, and these factors could possibly have influenced the outcomes of the study.

With regard to the finding of a greater reduction in ED attendances in implementation sites than in comparator sites, we cannot exclude the possibility that this result arose by chance. A recent systematic review found only one study that considered the impact of COPD care bundles on ED attendances and found no effect.^12^

These findings benefit from the pragmatic study design and robust analysis of detailed data from an extensive range of hospitals in England and Wales, enabling assessment of the delivery of care bundles in a ‘real-life’ care delivery context. Using a mixed-methods approach has ensured both the perception and practice of implementation have been evaluated. The findings are not necessarily due to lack of effectiveness of COPD bundles; rather they are likely due to lack of effective implementation of COPD bundles. This does not limit the importance of the results but highlights the ‘real life’ challenge of incorporating improvement science methods alongside reliable implementation of standard processes.

Future research should aim to address the temporality of QI practices through a longitudinal study offering greater and indepth analysis of the QI life cycle. Closer monitoring of implementation reliability would also be beneficial, ensuring that any observed lack of effect is related to the efficacy of the intervention rather than failure of implementation. The fields of improvement science and implementation research offer insights into more effective ways of facilitating QI in the NHS.^25^ Using these to ensure that QI efforts are as evidence-based as the best practice they seek to implement would enable future QI projects to make changes in the most effective way.

In summary, COPD care bundles had little impact on patient outcomes but appeared to be associated with a reduced number of subsequent ED attendances. The distinction between bundle implementation and standard care was not clearly defined and fidelity with care bundle delivery was generally very low, although care bundles had some impact on improving process reliability. These findings are unsurprising given the low level of bundle uptake in implementation sites, and it remains to be determined if COPD care bundles affect patient care and outcomes when they are effectively implemented. Healthcare professionals value care bundles, but despite the simplicity of the approach they are complex to implement, and clinicians require support to successfully change care pathways.
