# Supplementary figures and images for: Evaluation of ‘care bundles’ for patients with chronic obstructive pulmonary disease (COPD): a multisite study in the UK
Source: BMJ Open Respir Res. 2019 May 30;6(1):e000425. doi: 10.1136/bmjresp-2019-000425 (PMC6561386; doi:10.1136/bmjresp-2019-000425)

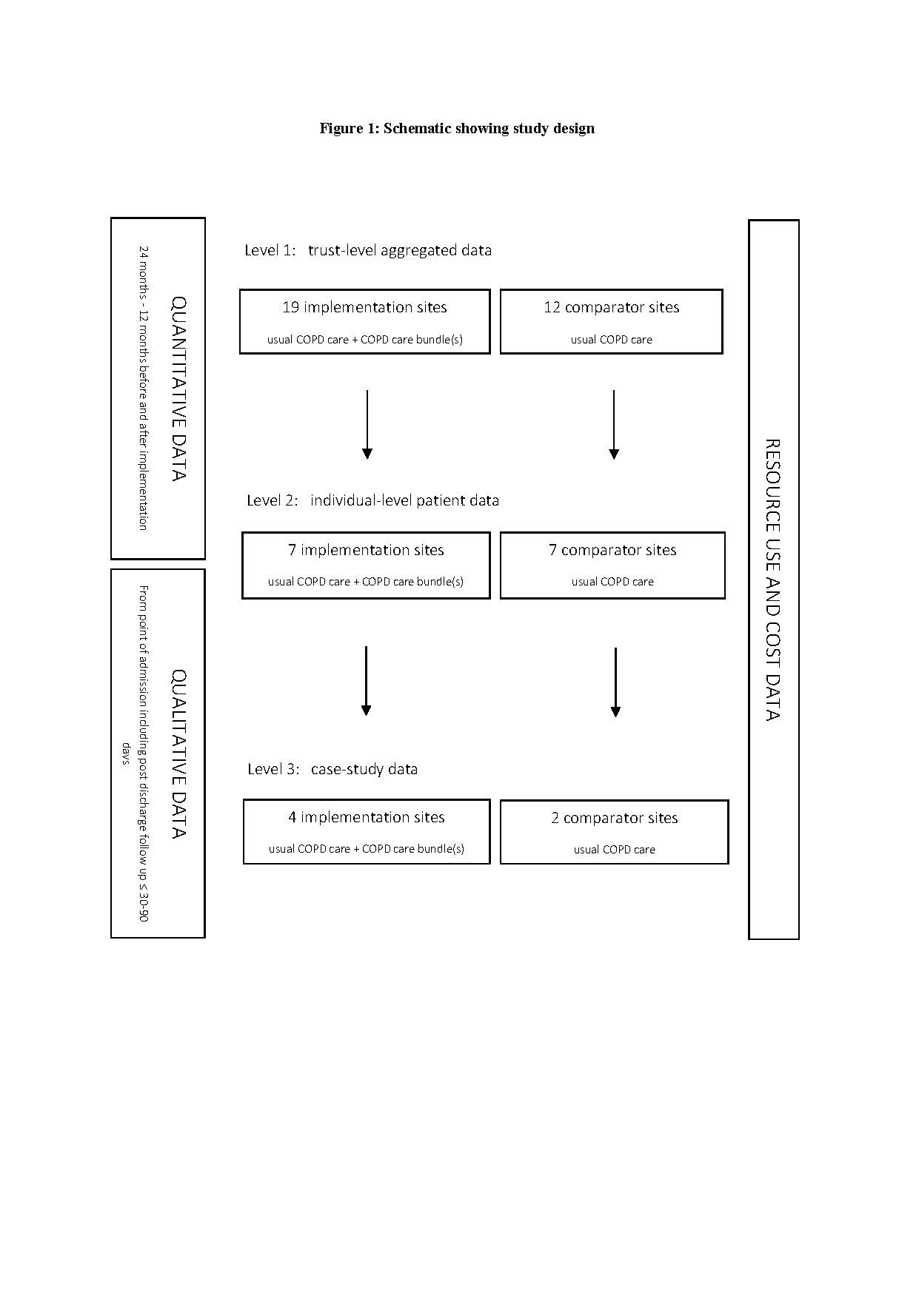

Supplement: Supplementary data [file bmjresp-2019-000425supp001.jpg]

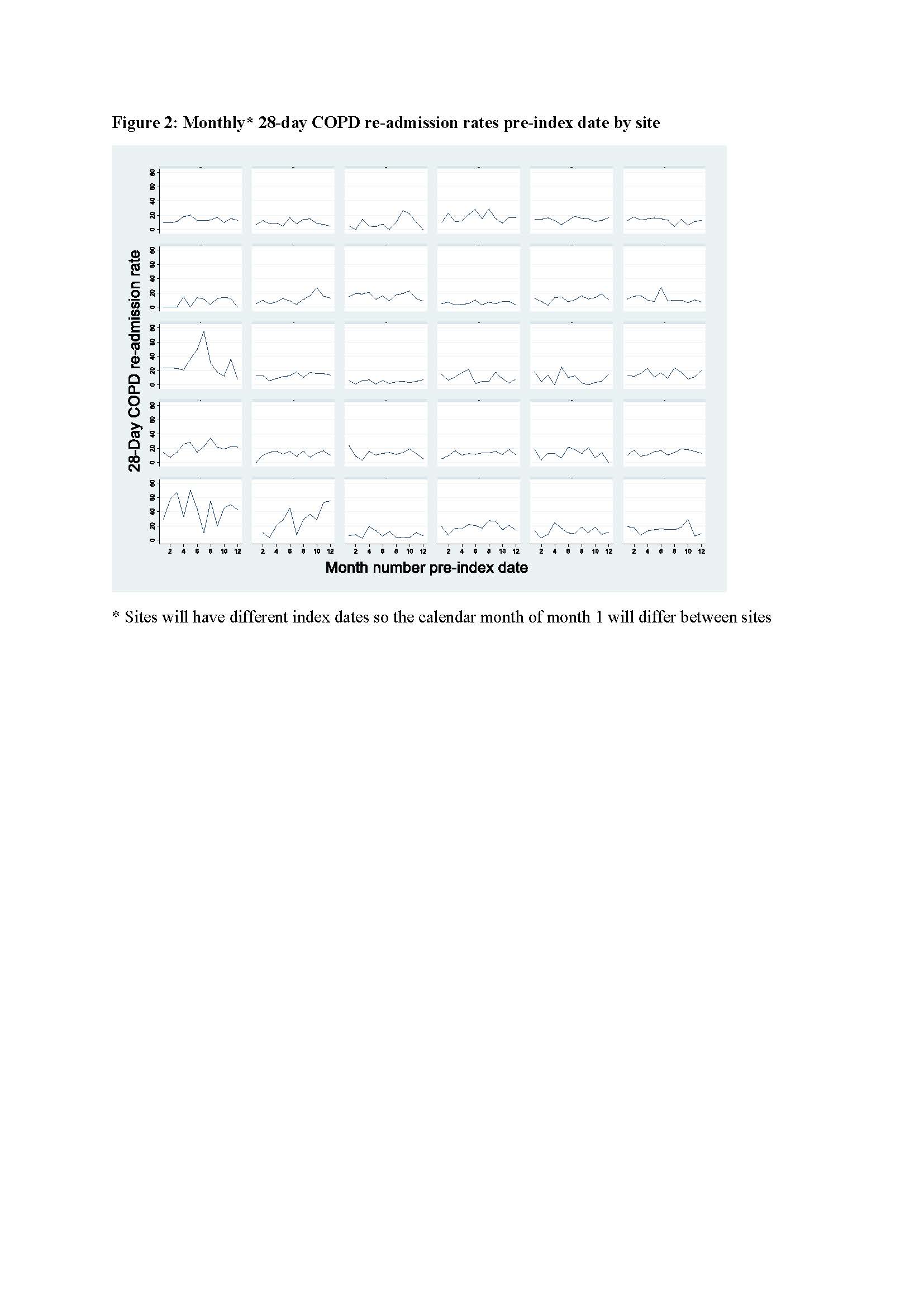

Supplement: Supplementary data [file bmjresp-2019-000425supp002.jpg]
